# EDUCATIONAL DISPARITIES IN COGNITIVE DECLINE AND DEMENTIA IN LATER LIFE

**DOI:** 10.1093/geroni/igad104.0001

**Published:** 2023-12-21

**Authors:** Pamela Herd

## Abstract

According to the most recent (2020) Lancet commission report, one of the most important determinants of Alzheimer’s Disease and related dementias (ADRD) is educational attainment. For those aged 80 and older, the risk for dementia is three times lower for those with a college degree compared to those without a high school degree, with even larger educational disparities by ethnicity. Despite these large and persistent gaps, there is much we do not understand about the relationship between education and dementia. Moreover, we have yet to develop effective clinical treatments that substantially slow or reverse the course of dementia. In this context, unpacking the education—ADRD relationship may be key to understanding how to delay ADRD onset. This panel explores how genetic risk, early life conditions, ranging from cognition and the quality of schooling to mid life factors, may mediate and moderate the relationship between education and cognitive decline and dementia in later life. The papers also pay close attention to variance by gender and ethnicity.

